# The quality of pre-announcement communication and the accuracy of estimated arrival time in critically ill patients, a prospective observational study

**DOI:** 10.1186/s12873-022-00601-z

**Published:** 2022-03-19

**Authors:** Michelle Maris, Sivera A. A. Berben, Wouter Verhoef, Pierre van Grunsven, Edward C. T. H. Tan

**Affiliations:** 1 Department of Emergency Medicine, Radboud University Medical Center, Nijmegen, The Netherlands; 2 Associate professor Emergency and Critical Health Care, HAN University of Applied Sciences Nijmegen, The Netherlands; Radboud University Medical Centre, Nijmegen, The Netherlands; 3 Emergency Medical Services Safety Region Gelderland-Zuid, Nijmegen, The Netherlands; 4 Trauma Surgeon/HEMS Physician/Chair Department of Emergency Medicine, Radboud University Medical Center, Nijmegen, The Netherlands

**Keywords:** Pre-announcement, Handover, Estimated time of Arrival, Observed Time of arrival, Emergency Department, (Helicopter) Emergency Medical Services

## Abstract

**Background:**

Efficient communication between (helicopter) emergency medical services ((H)EMS) and healthcare professionals in the emergency department (ED) is essential to facilitate appropriate team mobilization and preparation for critically ill patients. A correct estimated time of arrival (ETA) is crucial for patient safety and time-management since all team members have to be present, but needless waiting must be avoided. The aim of this study is to investigate the quality of the pre-announcement and the accuracy of the ETA.

**Methods:**

A prospective observational study was conducted in potentially critically ill/injured patients transported to the ED of a Level I trauma center by the (H)EMS. Research assistants observed time slots prior to arrival at the ED and during the initial assessment, using a stopwatch and an observation form. Information on the pre-announcement (including mechanisms of injury, vital signs, and the ETA) is also collected.

**Results:**

One hundred and ninety-three critically ill/injured patients were included. Information in the pre-announcement was often incomplete; in particular vital signs (86%). Forty percent of the announced critically ill patients were non-critical at arrival in the ED. The observed time of arrival (OTA) for 66% of the patients was later than the provided ETA (median 5:15 min) and 19% of the patients arrived sooner (3:10 min). Team completeness prior to the arrival of the patient was achieved for 66% of the patients.

**Conclusions:**

The quality of the pre-announcement is moderate, sometimes lacking essential information on vital signs. Forty percent of the critically ill patients turned out to be non-critical at the ED. Furthermore, the ETA was regularly inaccurate and team completeness was insufficient. However, none of the above was correlated to the rate of complications, mortality, LOS, ward of admission or discharge location.

**Supplementary Information:**

The online version contains supplementary material available at 10.1186/s12873-022-00601-z.

## Background

Effective communication between prehospital and in-hospital healthcare professionals in the emergency care pathway (the helicopter emergency medical services (HEMS), EMS and the emergency department healthcare professionals) is essential in order to guard patient safety of potentially critically ill or injured patients with life-threatening vital parameters [1]. The handover of a patient between healthcare professionals has been identified as a high-risk activity regarding patient safety, and is a common cause of information loss [2, 3], especially for the more critically ill or injured patients [4]. In order to standardize the structure of handover practices, various guidelines and training sessions have been implemented amongst EMS and ED personnel, although research has shown that there is still room for improvement [5–8].

Prior to the arrival of a potentially critically ill or injured patient at the ED, the triage nurse receives a pre-announcement from the (H)EMS professional. In the pre-announcement, information on the estimated time of arrival (ETA) of the patient at the hospital and about the patient’s condition is provided, according to the either widely used MIST (mechanism of injury; injuries found and suspected; signs; treatment given [9]) or SBAR (situation; background; assessment; recommendation) acronym. Based on the prehospital information about the patient’s condition, the ED staff decides whether to activate a basic or an advanced multidisciplinary (trauma) team in order to rapidly facilitate the patient’s resuscitation, diagnostic pathway and treatment upon arrival at the hospital.

Incomplete or inaccurate information in the pre-announcement or non-communicated changes in the patient’s condition during transport may result in a suboptimal preparation of the ED team, and potentially could compromise the patient’s safety. On the other hand, it could lead to an overestimation of the severity, causing redundant preparation at the ED, potentially leading to (unnecessary) multidisciplinary team activation and withholding care from other patients. Thus, accuracy of triage, based on the pre-announcement, affects patient safety [10]. If the observed time of arrival (OTA) is sooner than the ETA, this can result in an incomplete multidisciplinary team on arrival. If the OTA is later than the ETA, this could cause needless waiting of the team members in the resuscitation room.

The quality of pre-announcement communication can be defined as the level of completeness of the structured patient information on the MIST and the correspondence of the ETA with the OTA. In other studies information of prehospital handovers (MIST) from (H)EMS to ED healthcare providers was often (84%) missing [11]. Efforts to enhance the adherence to the handover guidelines, using e-learning programs, did not show significant improvement in information completeness [12]. Improvement in handovers could probably be achieved in other areas, such as clear documentation of the handover and the completeness of the receiving multidisciplinary (trauma) team at the ED.

Studies that have looked at the correspondence of the ETA with the OTA showed that EMS personnel often underestimate their travel time [13–16]. One study even concluded that 81% of the ambulances arrived later than the ETA, even for those with relatively short transport times [14]. However, these two studies were performed at the end of the previous century [14, 15], and circumstances have probably changed since then. Furthermore, they were conducted in countries with a lower population density/km [2] than the Netherlands and covered larger countries and districts.

The aim of this study is to describe current practice and the quality of the pre-announcement communication between (H)EMS and ED professionals of the region of a Level I trauma center in the Netherlands. More precisely, the study identified whether the OTA was congruent with the ETA in critically ill or injured patients arriving to the ED, and whether a multidisciplinary team was complete on arrival of the patient at the ED, in order to identify potential room for improvement in (pre-)hospital communication. Furthermore, we looked at the effects of above-mentioned parameters on mortality, length of stay (LOS) and in-hospital complications.

## Methods

The study had a prospective observational design and was conducted at the ED of the Radboud University Medical Center (Radboudumc), a large level I trauma center in eastern Netherlands, supplying care for 1.5 million inhabitants. In this center the availability of a multidisciplinary team is present 24/7. The setting included one HEMS and four EMS organizations. EMS services in the Netherlands employ registered nurses with several years of experience in emergency care, intensive care, or cardiac care. The HEMS crew is physician-staffed with a consultant in anesthesiology or trauma, assisted by a HEMS nurse and a pilot. Most of the time, a HEMS physician will travel with the EMS personnel by ambulance to the hospital due to relative short transport times in the Netherlands. Therefore, no difference between HEMS or EMS patients is made in the data.

### Inclusion- and exclusion criteria

All potentially critically ill or injured patients, of all ages, with a variety of injuries or diseases, who were prehospital triaged with a high level of urgency and transported to the ED by (H)EMS were included. The pre-announcement of the patient also had to be between 12:00 and 22:00 (see Additional file 1: appendix A). Self-referred potentially critically patients (not presented through the dispatch center) and critically ill patients who were pre-announced to the ED before 12:00 and after 22:00 were excluded. Patients who were presented to the ED by the EMS with non-emergency transport were also excluded.

### Study procedure

During an eight-month period from October 2017 until May 2018 research assistants were present at the ED during the previous mentioned time frame. They received a special training before the start of the study, including a theoretical instruction. Also, the first 2 days of observation were performed together with one of the researchers. They documented the information from the handover form, containing patient characteristics, cause of injury or illness, the MIST or SBAR, the ETA and treatment given (see Additional file 3: appendix C). Furthermore, they registered the arrival time of the patient, the individual team members, and the length of stay at the ED using a stopwatch. Hospital charts were checked to complete all data including outcome of all included patients. Due to the availability of research assistants the observation time was limited during daytime.

### Variables under study

The MIST was defined to be complete if information about the MIS was written down on the ED handover form, and information about treatment given (T) was provided in the ambulance patient care report form (which is part of the electronic patient record (EPR)). Information about the MIST was cross-checked with all data from the ambulance form. We defined the ETA to be corresponding to the OTA if the time difference between the two was less than 1 minute. A basic or advanced multidisciplinary team was complete if all mandatory members (disciplines) of the team were present in the ED resuscitation room (Additional file 2: Appendix B). The mandatory members of a team are considered as essential for the initial resuscitation and are highlighted with a ‘*’ in Additional file 2: Appendix B. Based on vital parameters and the diagnosis at the ED we decided if a patient was truly critically ill or injured upon arrival at the hospital.

### Sample size

The sample size was calculated based on a global estimation by the ED staff of the difference between team completeness and arrival time of the patient. We calculated we should include 222 patients.

### Analysis

All data were statistically analyzed using IMB SPSS Statistics 25. We used data of all the included patients for the analysis; missing data were not included in the analysis. Therefore, the number of included patients can vary for different variables/outcomes. The study was conducted in accordance with the Declaration of Helsinki and the medical ethical committee waived the need for review (number 2017–3797).

## Results

In total, 193 potentially critically ill or injured patients were included in the study. The mean age of patients was 51 years (SD 25 years), and most patients were men (58%). The most frequent cause of illness was trauma (32%), followed by cerebral diseases (30%) and pulmonary diseases (8%). For detailed demographics see Table 1.

**Table 1:** Characteristics of the included patients (*n* = 193)

Variable	Result
**Age** (years ± SD)	51 ± 25
**Gender**	
Male (number / percentage)	111 (58%)
Female (number / percentage	82 (42%)
**Cause of injury/illness (number / percentage)**	193 (100%)
- Trauma^a^	62 (32.1%)
- Intoxication (e.g. (auto-) intoxication with drugs)	12 (6.2%)
- Pulmonary disease (e.g. pneumonia, pulmonary embolism)	16 (8.3%)
- Cardiac disease^b^	11 (5.7%)
- Aneurysm (thorax/abdomen)	3 (1.6%)
- Cerebral disease^c^	57 (29.5%)
- Abdominal disease^d^	14 (7.3%)
- Gynecological disease (placenta praevia)	1 (0.5%)
- Other^e^	9 (4.7%)
- Unknown	8 (4.1%)

^a^Trauma: injury caused by trauma (e.g. (traffic/industrial) accidents, suicide attempts (excl. intoxications), burns;); ^b^cardiac (e.g., arrhythmias/cardiac resuscitation, congestive heart failure); ^c^cerebral (e.g., cerebrovascular accidents, subarachnoid hemorrhage, epilepsy; ^d^abdominal (e.g., gastro-enteritis, non-traumatic perforations, urosepsis/urinary tract infections, intussusception); ^e^other: hypothermia, anaphylaxis, non-specific complaints

### Quality of prehospital pre-announcement and MIST completeness (*n* = 193)

#### Mechanism of injury

For 192 patients (99%) information about the ‘mechanism of injury’ was provided to the ED.

The ED staff received no pre-announcement for one patient. In this case, it was a German HEMS that provided patient care and they arrived without notifying the ED.

#### Injuries found and suspected

For 86% of the patients (*n* = 165) information about injuries found and suspected (for trauma patients) or working diagnosis (for non-trauma patients) was noted in the observation form.

#### Vital parameters

For 15% (*n* = 28) of the patients the ED staff received information on respiratory rate, saturation, heart rate, blood pressure/systolic blood pressure, and the AVPU/GCS. For 86% (*n* = 165) of the patients, the ED staff did not receive complete information on vital parameters (Table 2).

**Table 2:** Percentage of completeness of vital signs in the pre-announcement by the EMS (*n* = 193)

Vital signs	Data complete *N* = 193
Respiratory Rate	30%
Saturation	70%
Systolic Blood Pressure	74%
Diastolic Blood Pressure	72%
Heart Rate	76%
AVPU / GCS -EMV	66%
Pupils	34%
Temperature	18%

#### Treatment given

For 70% (*n* = 131) of the patients, the provided prehospital treatment was known. For 12% (*n* = 24) the information on provided prehospital treatment was lacking in the ambulance handover form, and for 20% (*n* = 38) of the patients the ED staff received no ambulance handover form of the patient.

Missing information of the above-mentioned parameters was not correlated with mortality, length of stay (LOS) at the ED, LOS at the hospital or discharge location (death, other hospital, nursing home or home). Furthermore, the amount of missing information was not correlated with admission on a higher care department nor with the rate of complications during admission. See Additional file 4: appendix D for statistical analysis.

### Accuracy critically ill or injured (*n* = 193)

One hundred ninety-three patients were announced to be critically ill or injured (see Additional file 1: appendix A) which caused the activation of a multidisciplinary team. On arrival at the ED only 115 patients (60%) were still marked as critical. Of these critical patients, 3% died at the ED, 60% went to either the Intensive Care Unit (ICU) or the High Care Unit (HCU). Of the patients who were marked as non-critical at the ED most (93%) were admitted to a normal ward or discharged from the hospital (Table 3).

**Table 3:** Admission ward of ED patients (*n* = 187)

	Death	ICU	HCU	Relocation to other hospital	Normal ward	Home
Critical (*n* = 109)	3 (3%)	47 (43%)	19 (17%)	10 (9%)	26 (24%)	4 (4%)
Non-critical (*n* = 78)	0	0	2^a^ (3%)	4 (5%)	38 (49%)	34 (44%)

^a^ Two children. We only have a HCU ward for children in the Radboudumc

### Comparison OTA versus ETA (*n* = 178)

In total, valid data were collected on the ETA and the OTA for 178 patients. The comparison between the OTA and ETA of the patients showed that the overall tendency was that the patient arrived later than expected in the ED, with a median arrival time of 5:15 min after the ETA. However, it was not uncommon that the patient arrived sooner than anticipated, which was the case for 19% of the patients (see Table 4). The expected ETA met the actual time of arrival in the ED for 15% of the patients.

**Table 4:** Comparison OTA versus ETA (*n* = 178)

ETA versus OTA	N (%)	Median (min:sec)	Q1 (min:sec)	Q3 (min:sec)
Overall	178 (100)	+ 03:05	−00:09	+ 06:48
OTA < ETA	33 (19)	−03:10	−04:49	−02:05
OTA = ETA (± 1 min)	27 (15)	–	–	–
OTA > ETA	118 (66)	+ 05:15	+ 03:06	+ 08:39

A discrepancy of the ETA with the OTA had no effect on the rate of complications, mortality, LOS at the ED, LOS at the hospital, ward of admission or discharge location.

### Team completeness (*n* = 168)

For 66% of the patients (*n* = 111) the teams were complete (calculated with *n* = 107: median 04:26 min, Q1–08:08 min, Q3–01:50 min) prior to the arrival of the patient in the ED, whereas for 34% of the patients (*n* = 57) the team was incomplete (calculated with *n* = 49: median 01:37 min, Q1 00:19 min, Q3 03:15 min).

## Discussion

This study analyzed multiple essential elements in the prehospital communication between (H)EMS and emergency healthcare professionals at a large level I trauma center. So far, little research has been conducted that takes all these elements into consideration. Our results showed that the prehospital pre-announcement by the EMS on potential critically ill or injured patients often lacked information, and that a team is regularly incomplete on arrival of the patient. However, none of the above-mentioned missing parameters were correlated with a worsened outcome for the patient.

The handover often lacked information. In particular, information on the vital signs remained incomplete (86%). Similar findings were described by Harmsen et al. (2016), who found that 84% of the patient information was incomplete in prehospital handovers [11].

Currently, the evaluation about the patient’s condition is based on the prehospital handover and is crucial for preparation in the ED [1, 17]. Misjudgment of the patient’s condition based on lacking or incorrect information may lead to either the activation of a larger multidisciplinary team than necessary or team that is too small for adequate resuscitation compared to the severity of the trauma. Nevertheless, in the event of missing information, the ED assumes the worst-case scenario, resulting in upscaling to an advanced multidisciplinary team, which is potentially unnecessary. In our study 40% of the announced critically ill or injured patients turned out to be non-critical at arrival in the ED. This might partially be explained by the adequate intervention of our highly trained (H)EMS personnel, but undoubtedly a percentage of these team activations were redundant, causing withholding of care from other patients in the ED. Several studies concluded that other non-critical patients are affected by trauma activation, such as longer time to evaluation by a medical doctor, diagnostics, and length of stay in the ED [18–20]. The American College of Surgeons Committee on Trauma recommended aiming for an undertriage rate of maximum 5% and an overtriage of maximum 25–35% [21]. Past years formal triage scales have been developed to accurately assess the patients‘condition in order achieve this percentage, although research has shown that the performance of these triage scales varies considerably [22, 23].

Notably, only one in six patients arrived at the ED (OTA) within a one-minute range of the estimated time (ETA). Most potentially critically ill patients (66%) arrived later with a median of more than 5 min and 19% arrived 3 min sooner. Neeki et al. (2016) concluded in a previous study that EMS personnel underestimated their travel time to the ED by a median of 9 min (*n* = 555) [13], and other studies have also reported an underestimation of the ETA [14, 15]. The difference between the ETA and OTA in our study is smaller than previous studies, possibly because travel distances in our study region were smaller. The authors recommend the development of a real-time data transfer system that can stream monitor- and GPS-data (for the calculation of the ETA) directly from the EMS provider on route to the ED. This could enhance reliable prehospital communication between the prehospital field and the ED. It could also facilitate prehospital communication between ED professionals and on-scene (H)EMS professionals, which may improve preparation for the patient in the ED, time-efficiency (i.e., reduction of administration), and, above all, patient safety.

General activation of a multidisciplinary trauma team, stroke team and pediatrics team improve patient outcomes significantly [24–26]. In more than 66% we had a complete multidisciplinary team on arrival of the patient. The question remains to what extent expertise of individual team members contributes to patient safety in the team approach. This question is supported by the fact that missing information in the pre-hospital handover, a discrepancy in ETA/OTA and absent team members did not correlate to the rate of complications, mortality, LOS, ward of admission or discharge location. As mentioned before, when short of information one assumes the worst. Moreover, other factors play an important role in the quality of the resuscitation, such as leadership, communication, training, structure, and seeking help [24]. The combination of compromising factors (incomplete MIST, early arrival, and missing team members) increases the risk of suboptimal care and might have a negative effect on patient safety. Therefore, it is essential to make an adequate estimation of the patient’s condition to be able to sufficiently prepare and to prevent overtriage. This can only be accomplished when the prehospital communication is valid, reliable, and adequate.

## Limitations

This study has several limitations. Our observers were not present 24 h a day; hence the sample size is limited. Nevertheless, we choose to have our observers present during peak time, in which more than 80% of all patients are seen. The observers worked voluntary, and we did not have funding to arrange continuous monitoring during the night. The second limitation is that human errors by the observers were inevitable; key moments and/or arrival times can occasionally be missed. However, we provided clear and strict rules for these time measurements, such as absolute restriction of retrospective estimations of arrival/notification (etc.) times. Furthermore, it was only allowed to include a patient when present from the absolute beginning, just one patient at a time and all observers received a special training before the start of the study. We had considered video recording all potentially critical patients arriving at the ED for research purposes, but this plan was revoked because of the strict privacy regulations and the new General Data Protection Regulation. Despite these limitations, due to the usage of observers and stopwatches, the measurements made are far more accurate than a retrospective search in an electronic medical record. Also, we didn’t include 222 patients from our sample size calculation; however, this was a global estimation that was originally meant for comparison for interventions in the future. Finally, the study was performed in one of the eleven Level I trauma centers in the Netherlands, which belongs in the top three of trauma centers in terms of handling critically ill or injured patients. So even if the sample group was quite small, it is representative due to the variety in severity of the illness/trauma in potentially critically ill or injured patients.

## Conclusion

Prehospital communication from (H)EMS professionals to the ED colleagues in potentially critically ill or injured patients often appeared to be incomplete, especially regarding vital signs. Furthermore, patients regularly arrived more than 5 min later than the ETA, and a relatively small number arrived earlier than expected. For two-thirds of the patients, the multidisciplinary trauma team was complete at the ED on arrival. Nonetheless, missing information in the pre-hospital handover, a discrepancy in ETA/OTA and absent team members did not correlate to the rate of complications, mortality, LOS, ward of admission or discharge location.

## Supplementary Information

**Additional file 1: Appendix A.** Inclusion criteria.

**Additional file 2: Appendix B.** Team members.

**Additional file 3: Appendix C.** Observation form.

**Additional file 4: Appendix D.** Analysis.

## Data Availability

The datasets used and/or analysed during the current study are available from the corresponding author on reasonable request.

## Abbreviations

AVPU: Acronym for ‘alert’, ‘verbal’, ‘pain’, ‘unresponsive’
ED: Emergency department
EMS: Emergency medical services
EMV-score: ‘Eyes-‘, ‘motor-‘, ‘verbal-score’
EPR: Electronic patient records
ETA: Estimated time of arrival
GCS: Glasgow Coma Scale
HCU: High Care Unit
HEMS: Helicopter emergency services
ICU: Intensive Care Unit
MIST: Acronym for ‘mechanism of injury’, ‘injuries found and suspected’, ‘signs’ and “treatment given”
OTA: Observed time of arrival
SBAR: Acronym for ‘situation’, ‘background’, ‘assessment’, ‘recommendation’
SBP: Systolic blood pressure
SD: Standard deviation
