# Supplementary material for: The quality of pre-announcement communication and the accuracy of estimated arrival time in critically ill patients, a prospective observational study
Source: BMC Emerg Med. 2022 Mar 19;22:44. doi: 10.1186/s12873-022-00601-z (PMC8933928; doi:10.1186/s12873-022-00601-z)
Supplement: Supplementary file 1 — Additional file 1: Appendix A. Inclusion criteria. [file 12873_2022_601_MOESM1_ESM.docx]

**APPENDIX A – INCLUSION CRITERA**

*Inclusion criteria:*

- Potentially critically ill or injured patients with a variety of injuries or diseases, who were pre-hospital triaged with a high level of urgency and who were pre-announced and transported to the ED by (H)EMS.
- The pre-announcement of the patient by the (H)EMS took place between 12:00 and 22:00.
- Patients of all ages.

We defined a patient as potentially critically ill or injured when:

- One or more of the following vital parameters were observed in a patient ≥18 years of age:
  - Saturation < 90%.
  - Level of consciousness: AVPU (‘alert‘, ‘verbal‘, ‘pain‘, ‘unresponsive‘): verbal, pain, unresponsive or Glasgow Coma Scale (GCS): EMV (‘eyes‘, ‘motor‘, ‘verbal-score‘) < 14.
  - Temperature < 35.0 ⁰C or > 38.5 ⁰C.
  - Respiration rate < 8/min or > 30/min.
  - Heart rate < 40/min or > 130/min.
  - Systolic Blood Pressure (SBP) < 90 mmHg or > 200 mmHg.
- The patient had a high-energy trauma.
- Pre-hospital: the patient received HEMS care.
- In hospital: an advanced multidisciplinary team for the initial assessment was activated for the patient (see Appendix A).
- For patients under 18 years of age we adjusted and corrected the observed abnormal parameters according to the standard criteria used for that age.
- The triage nurse or emergency physician classified the patient as potentially critically ill or not, based on the prehospital announcement by the EMS to the ED.
