# Supplementary material for: The quality of pre-announcement communication and the accuracy of estimated arrival time in critically ill patients, a prospective observational study
Source: BMC Emerg Med. 2022 Mar 19;22:44. doi: 10.1186/s12873-022-00601-z (PMC8933928; doi:10.1186/s12873-022-00601-z)
Supplement: Supplementary file 2 — Additional file 2: Appendix B. Team members. [file 12873_2022_601_MOESM2_ESM.docx]

**APPENDIX B – TEAM MEMBERS**

| Team | Members |
| --- | --- |
| Basic team | Emergency physician* (team leader)  Emergency nurse*  Diagnostic radiographer |
| Trauma team | (Orthopedic) trauma surgeon (team leader)*  Emergency physician*  Emergency nurse*^#^  Surgery resident/junior doctor in surgery  Anesthesiologist or anesthesia resident*  Anesthesia nurse*  Radiologist or radiology resident  Diagnostic radiographer  Intensive Care Physician |
| Neurotrauma team | See members of the trauma team* + extra:  Neurologist or neurology resident or junior doctor in neurology*  Neurosurgeon or resident neurosurgeon^^^ |
| Cardiac resuscitation team | Emergency physician* (team leader)  Emergency nurse*^#^  Anesthetist or anesthesia resident*  Anesthesia nurse*  Cardiologist or cardiology resident*  Coronary-care-unit-nurse*  Intensive Care Physician |
| Thrombolysis team | Neurologist or neurology resident or junior doctor in neurology* (team leader)  Emergency physician  Emergency nurse*  Radiologist or radiology resident  Diagnostic radiographer |
| Paediatrics team | Pediatrician or resident pediatrician*  Emergency physician^^^  Emergency nurse*^#^  Pediatric anesthesiologist or anesthesia resident*  Anesthesia nurse*  Neurologist or neurology resident or junior doctor in neurology^^^  (Orthopedic) trauma surgeon^^^ |
|  |  |

* represent the mandatory team members, others team members are optional; ^#^ a minimum of two emergency nurses are required in the team; ^^^ dependent on the cause of injury/illness these clinicians are summoned to join the multidisciplinary acute care or trauma team.
