# Supplementary material for: The quality of pre-announcement communication and the accuracy of estimated arrival time in critically ill patients, a prospective observational study
Source: BMC Emerg Med. 2022 Mar 19;22:44. doi: 10.1186/s12873-022-00601-z (PMC8933928; doi:10.1186/s12873-022-00601-z)
Supplement: Supplementary file 3 — Additional file 3: Appendix C. Observation form. [file 12873_2022_601_MOESM3_ESM.docx]

OBSERVATION FORM Name observer:

Date: Diagnosis ambulance:

Age:

Gender: Diagnosis ED:

Patient number:

Critical patient YES/NO

| Time:  0:00,00 | PRE-NOTIFICATION EMS  Clock time: | | | | | | | | |
| --- | --- | --- | --- | --- | --- | --- | --- | --- | --- |
|  | FIRST PREHOSPITAAL VITAL PARAMATERS  Resp:  Sat:  BP:  HR:  Estimated Time of Arrival (ETA): | | | | AVPU:  EMV:  Pupils:  Temp: | | | | |
| Time: | ACTIVATION EMERGENCY PAGERS | | | | | | | | |
| Time of team completeness: | ARRIVAL INDIVIDUAL TEAM MEMBERS  (Neuro)trauma team | |  | Thrombolysis team | | | | |  |
|  |  |  |  | *Team member* | | *Time of arrival* | | |  |
|  |  |  |  | Emergency doctor | |  | | |  |
|  | *Team member* | *Time of arrival* |  | Emergency nurse | |  | | |  |
|  |  |  |  | Neurology resident/junior | |  | | |  |
|  | Emergency doctor |  |  | Radiologist/resident | |  | | |  |
|  | Emergency nurse |  |  | Radiographer | |  | | |  |
|  | Surgery resident/junior |  |  | Cardiac resuscitation team | | | | |  |
|  | Trauma surgeon or orthopaedist |  |  |  |  |  |  |  |  |
|  |  |  |  | *Team member* | | | *Time of arrival* | |  |
|  | Anaesthesiologist |  |  | Emergency doctor | | |  | |  |
|  | Anaest-resident |  |  | ED-nurse/CCU-nurse | | |  | |  |
|  | Anaest-nurse |  |  | Anaesthesiologist | | |  | |  |
|  | Radiologist/resident |  |  | Anaest-resident | | |  | |  |
|  | Radiographer |  |  | Anaest-nurse | | |  | |  |
|  | Intensivist |  |  | Cardiologist/resident | | |  | |  |
|  | (Neurology res/junior) |  |  | Intensivist | | |  | |  |
|  | (Neurosurgery-resident/junior) |  |  | Paediatrics team | | | | |  |
|  | Basic team |  |  | *Team member* | | | | *Time of arrival* |  |
|  |  |  |  | Emergency doctor | | | |  |  |
|  | *Team member* | *Time of arrival* |  | Emergency nurse | | | |  |  |
|  |  |  |  | Paediatrics resident/junior | | | |  |  |
|  | Emergency doctor |  |  | Paediatrician | | | |  |  |
|  | Emergency nurse |  |  | Paedr anaesthesiologist | | | |  |  |
|  | Radiographer |  |  | Peadr anaest-resident | | | |  |  |
|  |  | |  | Paedr anaest-nurse | | | |  |  |
|  |  | |  | Neurologist/res/junior/trauma specialist* | | | |  |  |
|  |  |  |  | *trauma surgeon or orthopaedist | | | | |  |
|  |  |  |  | | | | | |  |
|  | TIME-OUT PROCEDURE  Start time-out:  End of time-out: | | | | | | | |  |
| Time: | ARRIVAL PATIENT (When the patient goes through the door of the resuscitation room) | | | | | | | |  |
|  | HANDOVER BY THE EMS (When the EMS personnel is speaking)  Start handover:  End of handover: | | | | | | | |  |
| Time of first measurement: | FIRST INTRAHOSPITAL VITAL SIGNS (directly after the patient is connected to the monitor inhospital)  Resp: AVPU:    Sat: EMV:  Pupils:  BP:    HR: Temp: | | | | | | | |  |
| Time: | COLLECTION OF BLOOD SAMPLES (when the first blood collection tube is being filled) | | | | | | | |  |
| Time: | BLOOD SAMPLES SENT TO THE LABORATORY (when the blood collection tubes leave the resuscitation room) | | | | | | | |  |
| Time: | SIGN-OUT (when the patient goes through the door of their room, to leave the ED: home, ICU/MC, CCU, operation theatre, normal ward) OR time of death | | | | | | | |  |

Optional

| Time of first conducted: | FIRST X-RAY EXAMINATION/DIAGNOSTIC STUDY (tally + note the time of each conducted examination/study)  Echo-FAST (=pelvis, abdomen left/right, pericardium)  X-thorax  X-pelvis  X-extremities  CT (when the patient goes through the door in the resus.room to the CT-scan)  Echocardiogram |
| --- | --- |
| Time: | ORDER OF BLOODPRODUCTS (By phone/EPIC) by the ED-doctor/ED-nurse  *What is ordered and how much? (tally)*  Packed red blood cell concentrate (pRBC)/erythrocytes/ery’s:  Fresh Frozen Plasma concentrates (FFP)/plasma:  Platelet concentrate/thrombocyte/trombo’s:  Massive Transfusion Protocol (MTP) (package 1 or 2)  In case blood from the ED-fridge, note time here: |
| Time: | ARRIVAL OF THE FIRST BLOOD PRODUCT |
| Time: | ADMINISTRATION OF THE FIRST BLOOD PRODUCT |

NRS-score

1.
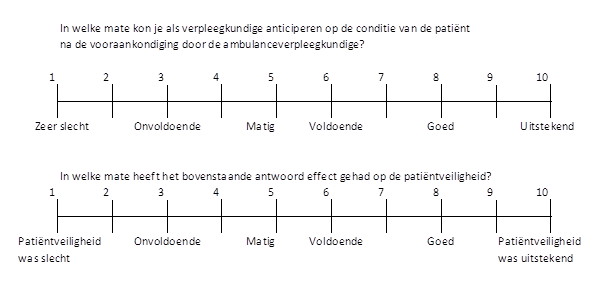
In which degree were you able to anticipate on the patient’s condition, based on the vital parameters from the prehospital handover by the EMS/HEMS? (for example, to decide which team to activate, to scale down or up and the preparing for emergency interventions)
2. In which degree has the above-mentioned answer influenced patient safety?


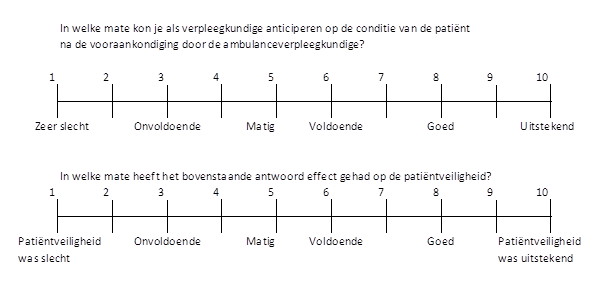


1. What grade do you give regarding to the quality of the resuscitation of the critical patient?


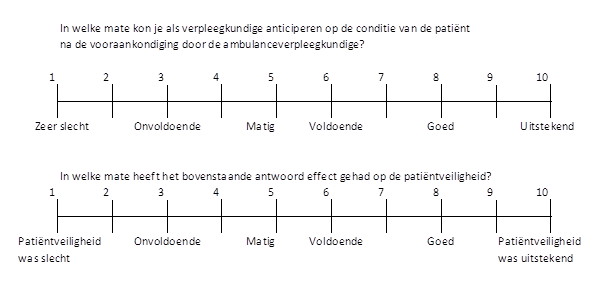


*question 1 and 2 are asked to the triage-nurse and question 3 to the ED-doctor in charge of the resuscitation.

Don’t forget the informed consent form!
