# Supplementary material for: The quality of pre-announcement communication and the accuracy of estimated arrival time in critically ill patients, a prospective observational study
Source: BMC Emerg Med. 2022 Mar 19;22:44. doi: 10.1186/s12873-022-00601-z (PMC8933928; doi:10.1186/s12873-022-00601-z)
Supplement: Supplementary file 4 — Additional file 4: Appendix D. Analysis. [file 12873_2022_601_MOESM4_ESM.doc]

**APPENDIX D STATISTICAL ANALYSIS**

Dependent variables: ‘complications’ and ‘alive at discharge’

Pearson's Chi square tests:

|  | Complications= yes | P-value chi-square test |  | Alive at discharge=no | P-value chi-square test |  |
| --- | --- | --- | --- | --- | --- | --- |
| Team complete |  | 0.81 |  |  | 0.89 |  |
| no | 10/56 (17.9%) |  |  | 5/57 (8.8%) |  |  |
| yes | 20/105 (19.4%) |  |  | 10/106 (9.4%) |  |  |
|  |  |  |  |  |  |  |
| Arrival patient |  | 0.89 |  |  | 0.87 |  |
| early | 5/33 (15.2%) |  |  | 2/33 (6.1%) |  |  |
| on time | 4/26 (15.4%) |  |  | 2/27 (7.4%) |  |  |
| late | 20/110 (18.2%) |  |  | 10/113 (8.8%) |  |  |

For the following variables the chi square tests were performed with respectively ‘complications’ and ‘alive at discharge’:

‘RR’, ‘saturation’, ‘SBP’, ‘HR’, ‘pupils’, ‘EMV or AVPU’ and ‘temperature’.

Also, for ‘Injuries found and suspected’ and ‘treatment given’.

None of the above-mentioned Pearson’s chi square tests had a p-value under 0.05.

The same conclusion was drawn from the analysis with ‘LOS ED’, ‘LOS hospital’, ‘ward of admission’, ‘discharge location’ as dependent variables.

The variable ‘mechanism of injury’ is not tested since almost everyone answered ‘yes’ (192/193).
